# BRFSS DATA ON COGNITIVE DECLINE AND CAREGIVING: NATIONAL AND STATE IMPLICATIONS

**DOI:** 10.1093/geroni/igae098.1556

**Published:** 2024-12-31

**Authors:** Erin Bouldin

**Affiliations:** University of Utah, Salt Lake City, Utah, United States

## Abstract

The Behavioral Risk Factor Surveillance System (BRFSS) is a state-based annual telephone survey conducted in all US states and three territories. BRFSS includes two optional modules that jurisdictions can use to understand cognitive decline and family caregiving. The Cognitive Decline Module collects information on self-reported changes in memory and thinking. This module offers value in understanding prevalence, with data from the 2016-2021 BRFSS suggesting that 1 in 9 people aged 50 years and older are experiencing subjective cognitive decline nationally. The module also offers the ability to analyze risk factors and health behavior related to detection of cognitive decline. The Caregiver Module collects information on prevalence of caregiving, relationship to the care recipient, the care recipient’s health condition, the duration and intensity of care, and caregiving tasks. Data from the 2016-2021 BRFSS show that 1 in 4 caregivers aged 50 years and older in the United States are caregivers for someone with dementia. This module offers value in understanding of caregiver health and health behaviors across a variety of demographics. This session will highlight recent publications and resources, including the Centers for Disease Control and Prevention’s publicly available Healthy Aging Data Portal from which these and other data can be analyzed and visualized. Data from these modules has also been used in innovative studies exploring risk factors for cognitive decline, cognitive changes among caregivers, and other findings. The session will demonstrate ways these data can advance the field at the national and state levels.

